# Uncovering Prolonged Grief Reactions Subsequent to a Reproductive Loss: Implications for the Primary Care Provider

**DOI:** 10.3389/fpsyg.2021.673050

**Published:** 2021-05-12

**Authors:** Kathryn R. Grauerholz, Shandeigh N. Berry, Rebecca M. Capuano, Jillian M. Early

**Affiliations:** ^1^Life Perspectives, San Diego, CA, United States; ^2^Department of Nursing, College of Arts and Sciences, St. Martin’s University, Lacey, WA, United States; ^3^Blue Ridge Women’s Center, Roanoke, VA, United States

**Keywords:** reproductive loss, miscarriage, perinatal grief, grief screening, abortion (induced), prolonged grief disorder

## Abstract

**Introduction:**

There is a paucity of clinical guidelines for the routine assessment of maladaptive reproductive grief reactions in outpatient primary care and OB-GYN settings in the United States. Because of the disenfranchised nature of perinatal grief reactions, many clinicians may be apt to miss or dismiss a grief reaction that was not identified in the perinatal period. A significant number of those experiencing a reproductive loss exhibit signs of anxiety, depression, or post-traumatic stress disorder. Reproductive losses are typically screened for and recorded numerically as part of a woman’s well-visit intake, yet this process often fails to identify patients emotionally troubled by a prior pregnancy loss.

**Materials and Methods:**

A summative content analysis of 164 recent website blogs from female participants who self-reported having experienced a miscarriage or abortion in their lifetime was conducted. The narratives were reviewed for details regarding the time span between the pregnancy loss and the composition of the blog post. The stories were analyzed for subsequent relationship problems and detrimental mental health conditions. Maladaptive reactions were contrasted for those that indicated a greater than 12 months’ time-lapse and those who had not.

**Results:**

More than a third (39.6%) of the women reported in the narrative that at least one year or more had passed since experiencing the miscarriage or abortion. For those women, the median time span between the loss and composing the blog was 4 years with a range of 47 years. Mental health conditions attributed to the reproductive loss by those who reported longer bereavement times included subsequent relationship problems, substance misuse, depression, suicidal ideation, and PTSD. The percent of reported maladaptive issues was more than double (136.9% vs. 63.6%) for those who reported that a year or more had passed since the loss of the pregnancy.

**Discussion:**

Grief reactions following the loss of a pregnancy may be prolonged or delayed for several months which can contribute to adverse biopsychosocial outcomes. Recognition and treatment of maladaptive grief reactions following a pregnancy loss are critical. Screening methods should be enhanced for clinicians in medical office settings to help identify and expedite the appropriate mental health assistance.

## Introduction

### Background

Research over the past two decades indicates that the emotional reaction or grief experience related to miscarriage and abortion can be prolonged, afflict mental health, and/or impact intimate or parental relationships. Maladaptive mental health sequelae associated with spontaneous abortion includes depression, anxiety, and post-traumatic stress (Hutti et al., 2015; Gold et al., 2016; Kaulathilaka et al., 2016; deMontigny et al., 2017; Farren et al., 2020). Research on mental health subsequent to early pregnancy loss as a result of elective induced abortions has historically been polarized, but recent research indicates an increased correlation to the genesis or exacerbation of substance abuse and affective disorders including suicidal ideation (Broen et al., 2005; Coleman, 2011; Steinberg and Finer, 2011; Bellieni and Buonocore, 2013; Sullins, 2016, 2019; Reardon, 2018). Several recent international studies have demonstrated that repetitive early pregnancy loss, including both miscarriage and induced abortions, is associated with increased levels of distress, depression, anxiety, and reduced quality of life scores in social and mental health categories (Kolte et al., 2015; Tavoli et al., 2018; Chen et al., 2020; Gao et al., 2020). Prolonged grief reactions related to other types of loss (spouse or older children) have been associated with poor mental and physical health outcomes including substance misuse, affective disorders, social and functional impairments, accidents, hypertension, cardiovascular disease, stroke, cancer, and suicidal ideation (Prigerson et al., 1997; Gerra et al., 2003; Hart et al., 2007; Lannen et al., 2008; Jones et al., 2010; Buckley et al., 2012; Nielsen et al., 2020).

A 2017 meta-analysis comparing the bereavement trajectories of different types of losses revealed that the prevalence of complicated grief reactions for perinatal loss was almost three times higher in comparison to other types of loss (Lundorff et al., 2017). In cohort and population based studies, intense perinatal grief reactions were shown to contribute to detrimental sequelae that included hypertension, weight gain, diabetes, heart problems, substance abuse, and increased risk of suicide (Dingle et al., 2008; Hvidtjørn et al., 2016; Lega et al., 2020). Overall, there is a paucity of research exploring the mental health concerns and related morbidity for women of childbearing age in primary healthcare settings, particularly in situations where racial or socioeconomic disparities are present (Marcus et al., 2003; Kim et al., 2010; Bowen et al., 2012; O’Hara and Wisner, 2014; Kingston et al., 2015; Shorter et al., 2021). Studies have recommended a standardized protocol for assessing the emotional well-being of women subsequent to reproductive loss by the primary provider(s) with follow-up visits or phone calls for several months after the loss occurred (Nynas et al., 2015; Farren et al., 2018). Studies evaluating the long term emotional impact of reproductive loss have generally been limited to 12 months and grief reactions delayed in onset are often not addressed.

Reproductive loss is common, occurring in a quarter of all pregnancies equating to approximately 2 million perinatal losses per year in the United States (Curtin et al., 2013; MacDorman and Gregory, 2015; Guttmacher Institute, 2019; Jatlaui et al., 2019). The grief experience related to reproductive loss, particularly for first trimester miscarriage and elective abortion, is often unacknowledged by society and healthcare providers (Lang et al., 2011; deMontigny et al., 2017; Bellhouse et al., 2018). When elucidating on disenfranchisement of the grief that can occur with reproductive loss, researcher Lang and her colleagues (Lang et al., 2011, p. 26) explained that, “Among healthcare professionals and society at large … perinatal loss is generally viewed as a less traumatic or prolonged experience that the death of an older child or an adult… [and] bereaved parents often find it hard to reconcile their intense feelings with society’s lack of validation.” Furthermore, researcher Farren and her colleagues (Farren et al., 2016, p. 8) said, “Exposure to early pregnancy loss on a daily basis may lead clinicians to normalize the experience and overlook the possible profound psychological sequelae.” Thus, regardless of the manner in which the loss took place, numerous women mourn their loss in secret and many reveal feelings of shame (Baxter and Akkor, 2011; Sisco et al., 2014; Duncan and Cacciatore, 2015; Bommaraju et al., 2016; Ebersole and Hernandez, 2016; Rafferty and Longbons, 2020). Consequently, disenfranchised grief compounded by the ambiguity inherent to first trimester losses can result in a grief trajectory that may be prolonged and complicated (Baxter and Akkor, 2011; Lang et al., 2011; Farren et al., 2020). Because of the disenfranchisement of reproductive grief, more women are turning to web-based reproductive bereavement resources (Capitulo, 2004; Geller et al., 2006; Hardy and Kukla, 2015; Rafferty and Longbons, 2020). The discretion and accessibility of computer mediated platforms are an outlet enabling anonymous disclosure of the reproductive loss experience and providing resources for many who feel their experience is otherwise socially dismissed (Capitulo, 2004; Klein et al., 2012; Ashford et al., 2016; Rafferty and Longbons, 2020). The proliferation of web-based supportive modalities provides the opportunity for research evaluating unrecognized gaps in reproductive healthcare (Jones and Alony, 2008; Denecke and Nejdl, 2009; Wilson et al., 2015; Litchman et al., 2019; Rafferty and Longbons, 2020).

### Objective

There are few longitudinal studies that evaluate the possible length of reproductive grief reactions or potential maladaptive mental health issues. A 2018 study showed that grief and depressive symptoms related to pregnancy loss can last up to 10 years (Kokou-Kpolou et al., 2018). That same study revealed a positive correlation between negative cognitions and both prolonged grief and depressive symptoms, thus identifying where cognitive behavioral therapy may yield benefit (Kersting et al., 2011; Bennett et al., 2012; Nakano et al., 2013; Kokou-Kpolou et al., 2018). Reproductive grief may not be acknowledged by care providers because it is not typically included in established care guidelines in the United States. The American College of Gynecology, in a 2018 practice bulletin regarding standards of care after early pregnancy loss, does not include addressing the emotional responses that some women may experience (American College of Obstetricians, and Gynecologists’ Committee on Practice Bulletins—Gynecology, 2018). Postpartum depression screening is recommended three to 12 months following the loss, but prolonged grief screening and emotional reactions beyond 3 months after the loss is not suggested in published standards of care (American College of Obstetricians, and Gynecologists’ Committee on Practice Bulletins—Gynecology, 2018). In this paper, we will examine both the length of time women may wait to disclose a reproductive loss from miscarriage or abortion, and the scope of subsequent responses to grief.

## Materials and Methods

The intent of this research study was not to generalize about reactions related to an early pregnancy loss, rather to determine the extent of some prolonged grief reactions that can occur, elucidate cited biopsychosocial outcomes, and uncover potential gaps in grief care provision. The research questions posed in this study for the narrative coding process included: (*RQ1) –* What is the length of time that women disclose regarding the date a miscarriage or abortion occurred and when they sought a venue to share their stories on a reproductive bereavement website blog post? (*RQ2*) *–* What, if any, maladaptive responses to reproductive grief were disclosed in the narratives commensurable with the time elapsed? The data was evaluated using summative content analysis which is a qualitative analysis process in which keywords or phrases, derived from the review of literature, are determined by the researcher before the data analysis, and then used to inductively interpret the contextual meaning of the narrative content (Hsieh and Shannon, 2005). The analysis included descriptive statistics to interpret and describe any evident practice gaps uncovered in the blog narratives.

**FIGURE 1:**
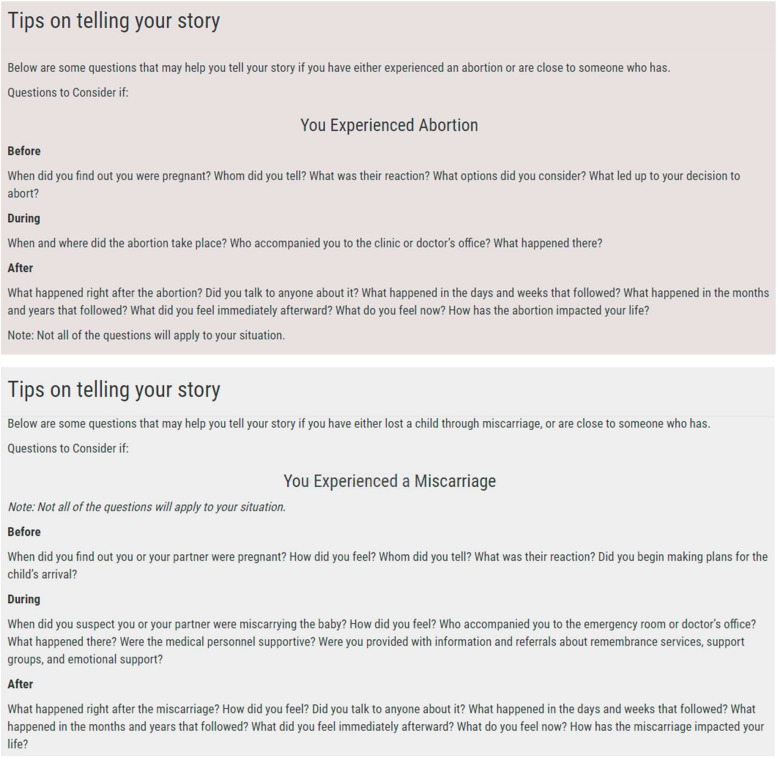
Informative captions available to the online users of abortionchangesyou.com^®^ and miscarriagehurts.com^®^ websites.

One hundred sixty-six narratives were collected from anonymous blogs posted on the stories page of the *miscarriagehurts.com* (MH) and *abortionchangesyou.com* (ACY) websites from November 4, 2019 to May 15, 2020. All of the narratives were downloaded from the websites’ administrative backend according to posting dates and supplied to the researchers by the websites’ facilitators. The reproductive grief websites that were utilized for the purpose of this study assure anonymity and offer some direction for those (men, women, and family members) wanting to divulge their personal story, but who are unsure how to compose their narrative (Figure 1; Rafferty and Longbons, 2020). Two of the narratives were eliminated from this study as they were focused on a family member’s experience of pregnancy loss (sister, grand-daughter), resulting in 164 total posts (ACY = 138, MH = 26). No men or partners of those physically experiencing the pregnancy loss had posted narratives during the selected sample time frame. The ACY website originated in 2008 and the MH website was launched in 2017 which may account for some of the difference in blog posting volume.

ACY and MH blog narratives were selected for this study because all posts are anonymous (a statement assuring protection of personal identifying information is posted on each website), bloggers do not interact with each other, creation of accounts are not required, the internet posted stories are unsolicited, and the publically posted blog narratives have been the subject of another recently published qualitative study (Rafferty and Longbons, 2020). Identification of the blog author’s name, web account addresses, ethnicity, and geographic location are excluded to maintain the privacy of the authors for the publically posted narratives. A third party privacy assurance organization oversees the secure connection and the privacy policies are posted on the “About Us” page on the website. Since the blog posts are posted anonymously and publically, the risks if any to the authors is minimal, therefore an ethical review was not required.

Data were interpreted by five doctorally or masters prepared nurses and mental health professionals. Additionally, the researchers have training in reproductive loss and grief care which consisted of an initial professionally accredited course and a 2 day in depth instructor course. The researchers all have professional experience assisting clients with reproductive grief and educating peers about perinatal bereavement.

Each narrative ranged from one sentence to three full pages. The individuals on the research team read the entire text of each story noting keywords indicating time elapses such as “months” or “years” as well as phrases including reports of the author’s age progression, birth(s) of subsequent child(ren), and obtaining a college degree. A total of 2 years was assigned for those who only indicated that “years” had passed or they had subsequent children. The principal author used a spreadsheet to determine a consensus regarding the length of time elapsed between the date when the blog was submitted and when the miscarriage or abortion occurred. Whole years were used for the descriptive data analysis. Partial years were rounded down to the nearest whole year indicated. For those narratives listing multiple losses, only the time elapsed since the most recent loss was accounted for in the analytics. In the 3 instances in which the latter loss was less than 12 months, 1 year was assigned for the statistical analysis because the investigators observed that the narratives were more focused on the losses with more lapsed time.

Maladaptive responses to miscarriages or abortions divulged within the narratives were recorded by 3 of the coders and then logged onto the spreadsheet by the principal author. The coding process also included noting in-text indications of subsequent affective, functional, or relational changes identified by keywords or phrases used by the narrative authors. Writers’ keywords such as *panic attacks*, *anxiety*, or *depression* and key phrases such as *started drinking more*, *had haunting nightmares*, and/or *flashbacks of the [event]* were evaluated as to the authors’ intentions and categorized by each of the researchers. Explicit disclosures of mental health issues, for instance in statements such as “I was diagnosed with PTSD” or “I had to drop my classes at school,” were also revealing of maladaptive reactions. Spreadsheet data of the identified reactions were evaluated by the principal author for inductive analysis. Basic descriptive statistics (range = *x*_*n*_−*x_1_*, mean = $\sum_{i=1}^{n}x_{i}/n$, median = *n* + 1/2 [odd] or *n*/2 [even]) were used to demonstrate the time span of many grief reactions and evaluate the scope of maladaptive responses to pregnancy loss in relation to time elapsed for the purpose of revealing a potential practice gap. The statistical analytics helped to discern an area of healthcare practice that is obscured by the current process protocols and the disenfranchised grief. Sandelowski et al. (Sandelowski et al., 2009, p. 210) exhibited that analytical data synthesis of qualitative research “allow[s] analysts to discern and to show regularities or peculiarities in qualitative data they might not otherwise see.”

## Results

### Elapsed Time Prior to Blog Disclosure

**FIGURE 2:**
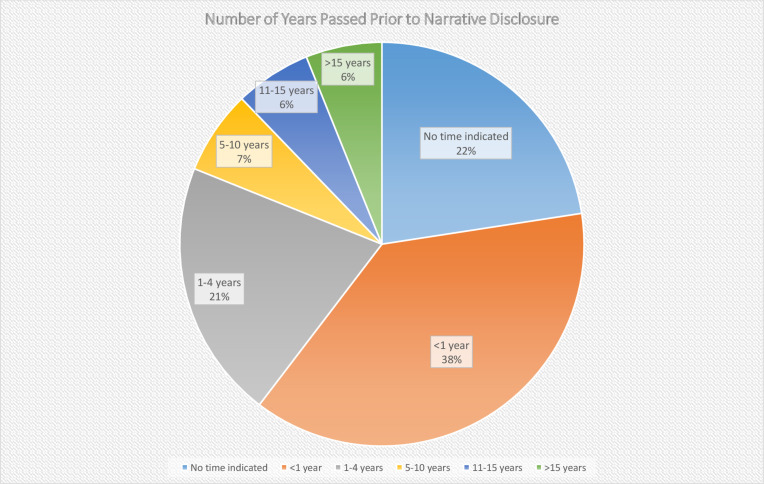
Representation of the indicated time elapsed between the pregnancy loss and blog post.

**FIGURE 3:**
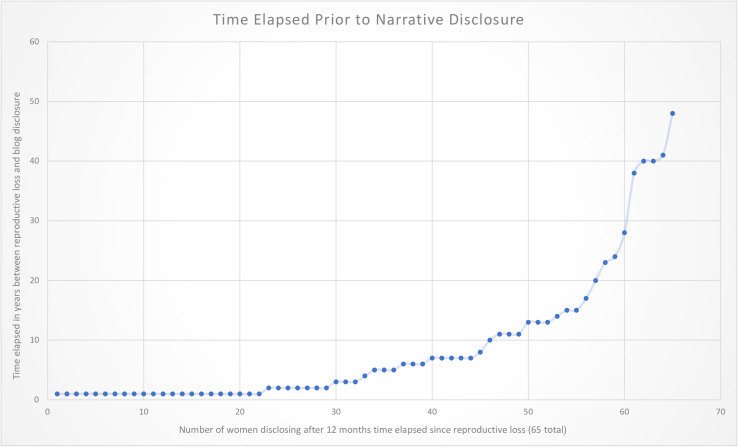
Time in years (≥1 year) that bloggers indicated between the pregnancy loss and the narrative composition.

Of the 164 reviewed blog narratives, 57.9% of those who had abortions and 73.1% of those who had miscarriages disclosed a time span that had elapsed since the pregnancy loss within the narrative. Sixty-five (39.6 %) of the women in the total sample indicated that their early pregnancy loss(es) occurred at least 1 year prior to composing their post (Figure 2). For those posting on ACY, 58 (42%) reported that the date of their induced abortion occurred a year or more ago; for those posting on MH, 7 (26.9%) of the women wrote that they experienced a miscarriage at least 1 year prior. The length of time for those posting about an abortion that occurred more than 12 months after the pregnancy loss ranged 47 years and the mean was 9 years with a median of 4.5; for those posting about their miscarriage, the range was 10 years and the mean was 3.3 years with a median of 1. When more than 12 months had passed between the pregnancy loss and the blog disclosure, analysis revealed that nearly half (49.2%) the women had waited more than 4 years to share their story (Figure 3). One woman who had experienced a miscarriage wrote about the passing of time and her grievance with the length and profundity of the experience, “Eleven years later I am still having issues. Not all the time but coming up to the anniversary [of the miscarriage]. April first is not funny at all. That’s the day that changed my life forever” [March 31, 2020]. One woman wrote the following in regard to the expanse of time that had passed since her abortion and her motivation to disclose it, “I am 63 years old. I had an abortion when I was 15 years old… All these years I have never spoken to anyone about this… And over the years I put it in the back of my mind, but it always seems to be there” [February 18, 2020].

Maladaptive mental health issues depicted by the women’s disclosures included intimate partner relationship strain, divorce, or break-up, distress in parental or subsequent child relationships, post-traumatic stress disorder, depression, suicidal ideation, anxiety with and without panic disorder, substance misuse, anorexia, and occupational dysfunction (Table 1). There were 15 reports of maladaptive relationship or mental health issues for the 37 women who did not indicate the time elapsed since the pregnancy loss in the narrative (5 women had multiple issues). There were 48 reports of maladaptive relationship or mental health issues for the 62 women who reported that less than 12 months had passed since the pregnancy loss (11 women had multiple issues). There were 89 reports of maladaptive relationship or mental health issues for the 65 women who reported that at least 12 months had passed since the pregnancy loss (26 women had multiple issues). The incidence (within the bounds of this sample) of reported maladaptive relationship or mental health issues more than doubled (136.9% vs. 63.6%) for those women who reported that one or more years had passed since their miscarriage or abortion.

**TABLE 1:** Number of years elapsed between pregnancy loss and blog post (0 = time elapsed not indicated) (< 1 = time elapsed less than 12 months).

	Time indicated	Anorexia	Anxiety	Depression	Suicidal ideation	PTSD	Substance Misuse	Intimate partner relationship	Relationships with children	Parental Relationships	Occupational adjustment	Time indicated	Anorexia	Anxiety	Depression	Suicidal ideation	PTSD	Substance Misuse	Intimate partner relationship	Relationships with children	Parental Relationships	Occupational adjustment	Time indicated	Anorexia	Anxiety	Depression	Suicidal ideation	PTSD	Substance Misuse	Intimate partner relationship	Relationships with children	Parental Relationships	Occupational adjustment	
	0											<1											1											
	0		x									<1							x				1											
	0											<1			x	x	x		x				1											
	0											<1			x				x				1											
	0											<1										x	1		x			x		x				
	0											<1											1											
	0				x							<1			x						x	x	1										x	
	0											<1									x		1							x				
	0											<1											1											
	0											<1											1			x	x							
	0											<1					x						1											
	0											<1											1			x		x						
	0											<1			x								1							x				
	0											<1					x						1			x				x				
	0											<1											1			x								
	0							x				<1					x						1		x									
	0											<1											1			x								
	0											<1					x						1											
	0											<1											1								x			
	0				x			x				<1							x				1			x				x				
	0							x				<1							x				1		x	x				x				
	0		x			x						<1											1							x				
	0				x		x			x		<1			x							x	2		x									
	0											<1		x			x						2			x				x				
	0			x				x				<1											2			x	x	x						
	0											<1											2		x	x		x		x			x	
	0											<1							x				2		x	x								
	0											<1							x			x	2					x		x				
	0											<1			x		x						2											
	0											<1											3											
	0											<1							x				3	x		x				x				
	0											<1											3							x				
	0											<1											4											
	0						x				x	<1									x		5						x	x				
	0											<1											5			x						x		
	0											<1											5							x				
	0											<1							x				6						x	x				
												<1			x				x				6											
												<1										x	6								x			
												<1			x				x				7					x		x				
												<1							x				7			x								
												<1							x				7									x		
												<1											7			x	x							
												<1					x	x					7								x			
												<1						x				x	8											
												<1											10											
												<1				x			x				10					x						
												<1											11				x			x				
												<1											11			x	x					x		
												<1											11					x						
												<1											13					x						
												<1											13	x				x		x				
												<1											13	x				x	x	x				
												<1								x			14			x				x				
												<1											15											
												<1											15	x		x	x		x	x			x	
												<1			x								17		x	x	x	x		x				
												<1											23											
												<1											24					x		x				
												<1											28											
												<1			x								38							x				
												<1							x				40											
																							40											
																							41							x				x
																							48											

*Maladaptive relational or mental health responses.*

### Maladaptive Partner or Parental Relationships

Forty-three of the women (26.2%) reported discord or dissolution of intimate partner relationships. This was the most prevalent report from the women composing narratives about their reproductive loss experience. Subsequent relationship changes were described in varying degrees ranging from discord to divorce to abuse. One disclosure recounted a slow drifting apart because of the miscommunication between the couple regarding the miscarriage and the misalignment of their emotional/grief reactions. She said in regard to the relationship, “We are still together. I thought that this time was going to be my chance to heal. But he never shared the hurt with me. And still till this day doesn’t…I literally cry alone almost every day a year later” [February 22, 2020]. Another woman recounted the abuse by her partner which escalated after the abortion, “I became really depressed, and miserable. Quiet and walking on eggshells so that I didn’t tick him off. We were also having financial issues at the time because he lost his job. It got worse. Screaming turned into being physical. Pushing me around, threatening to throw things at me, raising his hands as if he was going to hit me” [December 16, 2019]. Of the 65 women who reported that their reproductive loss occurred more than a year prior to composing their story, 24 (36.9%) reported that they experienced intimate partner discord or dissolution.

Parental or familial relationship discord was only reported for those blogging about their abortion experiences. Seven women (4.3% of the total) reported a strain in their relationship with parents. Four of the women who reported parental relationship strain also revealed that they were teenagers at the time of the loss, and half of that number indicated feeling pressured by their parents to have the abortion. One said, “I suffered for 11 years with anger, bitterness, and un-forgiveness toward my dad, who took me [for the abortion], which was the hardest thing” [March 25, 2020]. In contrast, two of the women in the abortion stories indicated relief and acknowledged their difficult situation was a significant experience that strengthened their relationships with others. Of the four posts written by teenagers, three disclosed that more than a year had elapsed since their abortion.

Tension in relationships subsequent to early pregnancy loss was attributed to the ambiguity of the loss, lack of acknowledgment, and support from loved ones evident in many of the ACY and MH narratives. One of the study bloggers poignantly wrote, “A miscarriage is hard because there is no funeral. There is no service of remembrance. There is no formal marking of a life passing. To me, that felt like there was no moving forward. I felt torn into pieces. My husband hurt, but no one else missed our baby” [January 17, 2020].

Four (2.4%) of the total number of narratives revealed strain or distress in relationships with subsequent child(ren) or being around others with children. One woman reported that she became pregnant with her son after a miscarriage which hindered her ability to grieve at the time because she was so busy with a newborn. She said, “It’s been almost 7 years [since the] miscarriage and I am still processing those difficult feelings. But I think am finally ready to acknowledge that life and name that child” [February 7, 2020].

### Subsequent Traumatic, Anxiety, and Affective Disorders

PTSD or post-traumatic stress symptoms like flashbacks of traumatic event or recurrent nightmares were mentioned in 22 (13.4% of the total) of the narratives. Fifty-seven percent of those indicating PTSD reported that more than 12 months had elapsed between the pregnancy loss and disclosure of that loss. Thirty-one percent of those indicating PTSD reported a time span greater than 10 years since their miscarriage or abortion, and two of those reporting PTSD in their narrative also disclosed that more 15 years had elapsed in their pregnancy loss. Only one woman who experienced a miscarriage reported PTSD; her loss occurred 11 years prior to sharing her story on the blog which may be indicative of the lasting impact that can result from a traumatic experience. She wrote, “I now have PTSD complete with flashbacks…Eleven years later I am still having issues” [March 31, 2020].

The nature in which the loss occurred and/or the required procedures were traumatizing for some women. The woman who experienced “PTSD complete with flashbacks” after the miscarriage disclosed that losing her baby in the toilet at the doctor’s office was “horrifying” for the healthcare professionals who attended to her. Another woman divulged how the surgical abortion procedure was the source of her trauma, “It traumatized me. I remember going in the actual room where the abortion was to take place and feeling scared. I looked around and saw a waste bin and thought to myself that I hope my baby wasn’t going to be in there… I remember before they put me to sleep that they put my legs on these poles and them spreading my legs wide open where it was cold. Then, next thing I knew I woke up and was just crying uncontrollably. I went to therapy but that didn’t help me at all. I did group therapy, tried talking to counselors, but nothing helped” [January 17, 2020]. Another woman recounted that it was the ultrasound images and her subsequent induced abortion that contributed to her distress, “I saw her in the ultrasound probe thing. She had a face, limbs, toes, and fingers. I wasn’t expecting her to be so formed yet. I’ll never get that image out of my head…For months I couldn’t be around babies without having a panic attack” [January 4, 2020].

Depression and depressed mood were frequently reported within the narratives subsequent to reproductive loss. Thirty (18.3%) of the total narratives reported a depressed mood or experienced significant depression. Twenty-seven were posted on the ACY platform and four were on the MH platform. Nineteen (11.6% of total) of the women who reported depression also noted that a year or more elapsed since the pregnancy loss (61.3% of those reporting depression).

Some indicated intense depressive symptoms and 12 (7.3% of the total) of the narratives included suicidal ideation. The MH narratives did not include suicidal ideation. Seven of those reporting suicidal ideation also disclosed that more than 12 months had elapsed since their abortion. One woman wrote about a limited period of anguish, “I had severe depression anxiety and suicidal thoughts for 2 weeks” [February 14, 2020] while others indicated more ongoing distress, “I think [about] suicide every day and cut myself on my arms, legs, chest, stomach, and sometimes wrist,” [April 10, 2020] or lasting struggles, “I cried for days, weeks, years. I suffered with depression. I was suicidal. If this was such a quick fix why did it hurt so much inside?” [February 25, 2020].

Many of the ACY or MH blog narratives included descriptions of fear and anxious feelings surrounding the event and recovery. Those who were afflicted with anxiety disorder and/or experienced panic attacks subsequent to the pregnancy loss were distinguished by the coders. Anxiety was a reported sequela in ten (6.1%) of the narratives. Seven indicated a time elapse greater than 12 months between the pregnancy loss and the post. One woman revealed how the anxiety subsequent to her loss impacted her, “I regretted my decision about 2 weeks later and sunk into extreme anxiety and panic attacks. It was hell. I was depressed. It has been a year” [March 29, 2020].

Four (2.4%) of the one hundred and thirty-eight women who wrote narratives on the ACY platform indicated anorexia subsequent to the pregnancy loss. Three of women also noted that more than 13 years had elapsed since the abortion, revealing the prolonged reaction to that experience. One woman disclosed enduring two induced abortions in the midst of severe emotional and physical abuse from both her intimate partner and mother, “I became very depressed, anorexic, getting down to 100 pounds for a 5’6 fully developed young adult” [January 10, 2020]. Her body mass index appeared to be dangerously low at 16.1. She continued to describe how she persevered and overcame her mental and physical anguish, obtaining a college degree. She was sharing her story to encourage others.

### Subsequent Substance Misuse

Substance misuse which included the disclosure of alcohol, cigarette smoking, and illicit drug misuse were indicated in nine (5.5%) of the narratives, five of whom also reported that a year or more had elapsed since the pregnancy loss. Only one woman in MH disclosed substance misuse as she tried to cope with her miscarriage saying, “I’ve started a cycle of homework, crying, caffeine, and drinking too much at night. I’m ignoring calls, emails, and friends. I struggle to get the motivation to do the simplest tasks. I don’t know how to fix myself” [November 26, 2019]. One woman wrote that her substance abuse problem worsened after having an abortion. She also disclosed that 9 years later when she “received the gift of sobriety,” her grief about the induced abortions became more intense requiring professional assistance. One woman reported that she turned to substance abuse as a means of coping with her self-loathing, pain, and grief experienced after an abortion. She said, “I hate myself. I don’t want to be here anymore. I started using drugs to cope with the pain” [April 4, 2020]. Another blogger described how the intensity of her grief fluctuated and she utilized alcohol as means to numb more intense emotional responses. She wrote, “Little things trigger my emotions about it now. Some days I think about it and can move right along. Other days I’m paralyzed and can’t leave my couch for the rest of the day. I drown myself in wine and books” [May 1, 2020].

### Maladaptive Occupational Adjustment

Difficulties achieving educational or occupational goals, absenteeism, and/or job losses were reported occurring subsequent to or exacerbated by the experience of the pregnancy loss. Eleven women (6.7%) reported occupational difficulties; four of whom composed their blog post one or more years after the pregnancy loss. One woman reported difficulty maintaining her academic aspirations after experiencing an early miscarriage recounting, “I dropped presidency of my 2 clubs temporarily, and I dropped a class, and I still am way behind but I hardly care” [November 26, 2019]. Another woman reported that her career working with children made coping with an abortion difficult and disclosed, “being a preschool teacher I begin finding myself not wanting to be at work.” Prolonged distress after an abortion contributed to one woman’s absenteeism. She said, “Its interfering with work. I’m taking days off, sleeping through my alarms so I’m hours late I can’t focus on anything and honestly I don’t care about anything anymore” [December 12, 2019]. One woman divulged how her intense desire to talk with anyone about her abortion experience impacted an employment opportunity. She said “I spent 2-3 years desperate for anyone to talk to or tell [about the abortion]. It even came up in a job interview… I was not hired, but the manager seemed to understand” [April 12, 2020].

### Impact of Multiple Pregnancy Losses

Eight women (4.9%) in the study sample wrote that they had experienced more than one pregnancy loss. Seven of the women had posted experiencing multiple losses on the ACY website. One woman reported that she had endured 3 miscarriages on the MH website. The woman who experienced multiple miscarriages wrote, “I feel broken. Like there’s something wrong with me or I’m doing something wrong. I feel like a horrible wife, because I know my husband shares my dreams of a big family. It hurts me so bad that I can’t give him that or give my daughter a sibling. I don’t know how to deal with it, because outside of parenting my child, I am no one…. I don’t want my depression and anxiety behind miscarriage to carry such a weight that I’m not able to be the mother and lover that I want to be” [April 28, 2020]. Seven of the women who reported multiple pregnancy losses also indicated intimate partner relationship discord or dissolution. Four of the women who had experienced multiple pregnancy losses also reported experiencing depression. Two women who had experience multiple pregnancy losses divulged PTSD related to those experiences and another reported how her substance abuse continued to escalate with each of her three abortions.

## Discussion

The results of the content analysis uncovered the emotional, relational, and grief related struggles that many women endure after experiencing a miscarriage or abortion. Some women grappled with maladaptive relationship and mental health issues related to the reproductive grief for years or even decades. The numbers of depression, anxiety, and PTSD reactions to pregnancy loss from this small sample study (164 total) are comparable to a larger quantitative study done in the United Kingdom which examined the emotional reactions of 1,098 women after a miscarriage or ectopic pregnancy published in 2020 (Farren et al., 2020). Ferran and her colleagues found that 15-25% of women experiencing a reproductive loss exhibit diagnosable signs of anxiety, depression, or post-traumatic stress disorder up to 9 months following the pregnancy loss (Farren et al., 2020).

Although there is no research specifically evaluating how the grief experience related to early pregnancy loss impacts the healthcare system or socioeconomic costs overall, a study by The Grief Recovery Institute in 2017 (based on projection from their 2003 study) estimated the annual cost of grief in the workplace was approximately $100 billion (James and Friedman, 2003; Genevro and Miller, 2010; Moeller, 2017). Unresolved or prolonged grief has the potential risk for affective disorders often related to detrimental co-morbidities and potential, extensive socioeconomic ramifications. According to the APA, the annual cost of depression approximated $210 billion from 2010-2012 (Greenberg et al., 2015). Depression can lead to increased healthcare utilization, higher costs, increased morbidity, and higher rates of co- or multi-morbidities (Greenberg et al., 2015). Depression contributes to an increased rate of heart disease and inflammatory auto immune diseases including: diabetes, arthritis, headaches, insomnia, and chronic pain (Halaris, 2013; Cohen et al., 2015; Karling et al., 2016; Miller and Raison, 2016).

Five percent of the women in this study reported misusing substances following their reproductive loss. Substance misuse has an overwhelming impact on both the healthcare system and society because of the prevalence and increasing detrimental effects. In 2010-2013, there was an estimated $740 billion economic costs attributed to crime, lost productivity, and healthcare expenses (NIH, 2020). The number of those in this study who indicated cigarette, alcohol, or illicit drug misuse was significant considering the potential deleterious long term health to the individual and the greater socioeconomic impact. In a longitudinal cohort study, the relative risk for substance abuse disorders doubled when there is a history of abortion regardless of pregnancy intention (Sullins, 2016). However, there is a paucity of research studying substance misuse in those who have experienced miscarriage.

Anorexia was another detrimental health problem subsequent to abortion for a handful of the women in this study. Eating disorders, specifically anorexia, can involve lengthy therapeutic treatment and increased mortality risks (Chesney et al., 2014). There was a calculated 6–12% higher risk for premature death in women with anorexia nervosa, with a 35–85% recovery rate with 5 or more years of recovery treatment (Lebow et al., 2018).

### Screening for Reproductive Grief

The incidence of prolonged and/or complicated reproductive grief reactions has been illustrated which demonstrates the importance of evaluating the grief for years after the loss. Prior to 2001, there were no methods to easily screen for a prolonged or complicated grief reaction for those with any type of loss in the primary care or outpatient healthcare setting. A Brief Grief Questionnaire (BGQ) was developed by Shear and Essock as a screening tool to identify persons suffering from complicated grief after the September 11, 2001 attacks (Shear et al., 2006). This screening tool utilizes five questions with three-point Lickert scale answers to assess for the extent of complications experienced in an individual’s grief experience. The BGQ has more recently been used to follow the course of grief reactions in military mental health (Delaney et al., 2017) and grief (unspecified losses) presented in an integrated primary/behavioral health care clinic (Patel et al., 2019). The screening method has been found to be a useful assessment tool assisting health providers to recognize grief and, if appropriate, refer a patient with prolonged grief symptoms for mental health services. These studies found the BGQ to be easily administered and effective in identifying grief responses that are more prolonged and complicated than more “typical” mourning reactions.

Reproductive losses are routinely screened for during well-women exams, yet there is no standard method of evaluating whether or not the losses are associated with prolonged grief or maladaptive responses. A 2017 study revealed that satisfaction with healthcare services at the time of the reproductive loss and subsequent follow up visits was integral for a healing grief trajectory (deMontigny et al., 2017). Failure to address an emotional reaction can result in disenfranchised grief and impair therapeutic patient/provider alliances as was the case in this woman’s account of her follow-up appointment, “my nurse walked in with a grin on her face, asking how I was before looking up to see my swollen eyes, then looking down at my chart as the color in her face quickly faded. She clearly did not know she was walking in on someone who had just lost their dream. Her first question to me was, ‘Would you like to get put on birth control?’ I sobbed, cried out, ‘What!’ and cried some more. How could her mind go straight to that??! I wanted nothing more than to still be pregnant” [March 15, 2020].

An initial screening question like, “Are you experiencing any distress related to pregnancy loss?” could be posed to the patient when a reproductive loss is noted on the intake. This question may procure an extemporaneous conversation between the patient and provider especially when one has harbored a prolonged emotional response or grief reaction for years or even decades. A patient’s confirmation of grief related to a pregnancy loss could be responded to with validated methods of screening for prolonged grief reactions with tools like the PG-13 (Zhang et al., 2006) which is 13 questions or the BGQ (5-questions) (Shear et al., 2006), but neither have been studied for applicability with reproductive losses. The Perinatal Grief Scale is widely utilized in mental health and research, but is 33 questions and geared for therapeutic interpretation rather than screening for necessity of further treatment or therapy in the primary medical office (Toedter et al., 1988, 2001). A shorter screening and assessment tools like the BGQ or PG-13 should be modified and validated for use in the outpatient setting to evaluate for the presence of prolonged or complicated grief.

### Limitations of the Study

Findings in this study are not generalizable because anonymous narratives were used which lack valuable sociodemographic information. It also is recognized that there is self-selection for those who choose to write their reproductive loss stories in narrative form. Participation in ACY or MH blog supportive activities indicates that their experiences were impactful enough to seek a venue to disclose very personal events in their life; whereas, others experiencing pregnancy loss may not be similarly impacted. Furthermore, the studied blog postings were drawn from only two websites with the same oversight source rather than from a number of independent sites. Qualitative analysis of the blogs’ content by the investigators was limited to interpretation which is inherent in all qualitative research. Five advanced practice nurses/mental health professionals conducted the study to maintain the integrity of the content interpretation and analytics.

Interestingly, half of the narratives were composed at the time of the COVID-19 pandemic. Some indicated that shelter in place mandates, fear of the illness, and/or their access to health care impacted their decision to voluntarily terminate a pregnancy. One woman said, “As I sit here in quarantine, I believe I did the right thing but I have so much pain. Since I have no one to share with that would understand, I am sharing it here to relieve some pain.” In 2020, there was a modest 13% increase use of the studied websites in comparison to previous years which may be indicative of the pandemic’s impact on pregnancy loss distress or a reduction in bereavement support offered by typical healthcare venues.

The fact that no partners had posted story blogs on the ACY or MH websites in the 6 month time period from which the analyzed narratives were drawn perhaps indicates that either those not physically experiencing the loss are impacted less, or they feel less entitled or apt to publically share their grief experience.

### Recommendations for Future Research

More qualitative and quantitative research on the grief reactions and mental health effects of women enduring early pregnancy loss should be done to explore methods of identifying and assisting those impacted after the miscarriage or abortion took place. Since our sample size for miscarriages was small (26 narratives), larger qualitative studies evaluating the experiences of women enduring miscarriage may provide further insights regarding possible gaps in the provision of care. Repetitive early pregnancy losses evaluated in this study revealed intense emotional reactions and intimate partner discord and/or dissolution consistent with recent findings in international studies and should also be studied domestically. There is a paucity of research studying relationship discord and the incidence of subsequent substance misuse in those who have endured miscarriage.

Future studies should evaluate and provide more information about the aspects of reproductive grief reactions in other populations. The impact of reproductive grief analyzed in this study indicated possible practice gaps related to early pregnancy loss grief assessment and evaluation in outpatient healthcare settings in the United States. Standards of care for reproductive grief care provision in other countries were not exhaustively explored and provides an impetus for future international research. Impacted partners’ or family members’ reactions to reproductive loss should also be considered for future qualitative and quantitative research studies.

## Conclusion

Early pregnancy loss whether miscarriage or abortion should be approached holistically. Prolonged or severe emotional reactions related to such loss has been evidenced in recent studies, but continues to be unacknowledged, underestimated, and, unfortunately, unaddressed (Nynas et al., 2015; Farren et al., 2020). Holistic care for those with a history of early pregnancy loss should include evaluating the emotional response to the loss, appraising reproductive grief, effectively assessing maladaptive responses, and initiating treatment at subsequent visits. Ongoing assessment and evaluation of distress related to pregnancy loss is necessary, as the reactions to the loss may be delayed or prolonged, potentially contributing to significant morbidity and mortality. It is imperative that the standard of care regarding the grief and emotional reactions related to early pregnancy loss is optimized with applicable screening modalities and evidenced therapeutic interventions. As one woman said regarding her pregnancy loss, “Thirteen years later and I am only peeling back the top, [or] maybe [the] middle layers of an onion to understand it and heal. I don’t believe the pain will ever stop.”

## Data Availability Statement

The original contributions presented in the study are included in the article/supplementary material, further inquiries can be directed to the corresponding author.

## Author Contributions

All authors listed have made a substantial, direct and intellectual contribution to the work, and approved it for publication.

## Conflict of Interest

The authors declare that the research was conducted in the absence of any commercial or financial relationships that could be construed as a potential conflict of interest.
